# Role of diffusion weighted imaging, apparent diffusion coefficient and magnetic resonence perfusion among acute ischemic stroke patient

**DOI:** 10.6026/973206300221887

**Published:** 2026-03-31

**Authors:** Sachi Mall, Sujeet Kumar Jain, Ashish Kumar Shukla, Mohit Gaur, Gaurav Khurana, Shipra Chaudhary, Ranjeet Singh

**Affiliations:** 1 Department of Radiodiagnosis, Santosh Medical College, Santosh Deemed to be University, Ghaziabad, Delhi NCR, India

**Keywords:** Acute ischemic stroke, diffusion-weighted imaging (DWI), apparent diffusion coefficient (ADC), MR perfusion, diffusion perfusion mismatch

## Abstract

Acute ischemic stroke requires quick identification of infarct core and salvageable tissue for proper management. MRI techniques, including diffusion-weighted imaging (DWI), apparent diffusion coefficient (ADC) mapping and MR perfusion, provide essential early physiologic information. A study conducted at Santosh Medical College Hospital involved 60 patients presenting within 24 hours of neurological symptoms. The results showed DWI and ADC reliably detected restricted diffusion, while MR perfusion highlighted perfusion abnormalities. The findings enhance stroke evaluation by demonstrating the importance of combining diffusion and perfusion imaging to better understand stroke pathophysiology, potentially improving patient outcomes.

## Background:

Acute ischemic stroke is a neurological emergency in which abrupt reduction of cerebral blood flow initiates a time-dependent cascade of metabolic failure, ionic pump dysfunction, excitotoxicity and progressive neuronal injury [1]. Because the probability of meaningful recovery decreases as irreversible infarction expands, modern stroke care prioritizes rapid, accurate diagnosis and early identification of patients who may benefit from reperfusion therapies [2]. Clinical examination alone cannot reliably distinguish irreversible infarct core from potentially salvageable tissue and early conventional imaging can be normal or nonspecific in the hyperacute period. As a result, MRI has assumed an important role in acute stroke pathways by providing physiologic and tissue-level biomarkers that support "tissue-based" triage rather than reliance solely on symptom onset time [3]. Diffusion-weighted imaging (DWI) is widely recognized as the most sensitive MRI technique for early detection of ischemia. Within minutes of arterial occlusion, cellular energy failure leads to cytotoxic edema and restriction of water diffusion, producing conspicuous signal hyperintensity on DWI [4]. However, DWI signal can be influenced by T2 shine-through and other confounders; therefore, ADC mapping is essential to confirm true restriction and improve diagnostic confidence. ADC provides a quantitative correlate of diffusion behavior, typically showing reduced values in acute infarction and evolving over time with pseudonormalization in later phases [5]. Together, DWI and ADC offer a robust method to identify infarct core, characterize lesion age when interpreted with other sequences and strengthen diagnostic certainty across a range of infarct sizes and locations. Technical developments, including the use of optimized and synthetic high b-values, can further improve conspicuity of subtle infarcts and help standardize lesion detection across scanners and protocols [6]. While diffusion imaging excels at defining infarct core, it does not by itself describe the full physiologic extent of ischemia, particularly the penumbra hypoperfused but potentially viable tissue that may be rescued with timely reperfusion. This limitation is clinically important because a patient may harbor a small diffusion-defined core but a larger territory of threatened tissue [7]. Contemporary stroke management therefore increasingly uses imaging to estimate tissue viability and to guide therapy in patients with unclear onset, wake-up stroke, or delayed presentation. In these scenarios, MRI-derived patterns such as diffusion-FLAIR mismatch and broader tissue-based criteria have supported safer selection for intravenous thrombolysis beyond strict time windows in appropriately chosen patients. Perfusion imaging complements diffusion by depicting cerebral hemodynamics and identifying regions of reduced blood flow or delayed perfusion that may extend beyond the diffusion lesion [8]. The practical concept of diffusion-perfusion mismatch emerged from the need to approximate salvageable tissue and support individualized decision-making. When perfusion deficit exceeds the diffusion-defined core, the mismatch is interpreted as potential penumbra, which can influence both acute treatment selection and prognostic assessment [9]. In addition, combining perfusion imaging with vascular imaging helps link tissue-level effects to upstream arterial pathology and collateral status, allowing a more comprehensive understanding of stroke mechanism and severity. This integrated approach is increasingly emphasized in contemporary stroke imaging practice, particularly for anterior circulation large vessel occlusion where rapid decisions regarding thrombectomy are required [10]. MR perfusion can be obtained using contrast-based techniques or non-contrast approaches such as arterial spin labeling (ASL). ASL is attractive because it requires no exogenous contrast, can provide quantitative cerebral blood flow estimates and is useful in patients with renal impairment, contrast allergy, or when repeated follow-up imaging is needed. In acute ischemic stroke, ASL can demonstrate hypoperfusion, collateral flow patterns and arterial transit artifacts that reflect delayed arrival and hemodynamic compromise [11]. A structured understanding of ASL patterns can improve interpretation consistency, aiding multimodal MRI strategies. Comprehensive MRI protocols combine various sequences to assess infarct core, hemodynamic compromise and vascular issues, aiming for streamlined, time-efficient imaging. As reperfusion therapies evolve, standardized imaging protocols and decision criteria are crucial for optimizing patient outcomes, particularly in late-window or unknown-onset cases. Ongoing evaluation ensures that imaging data supports consistent clinical decisions [12]. Therefore, it is of interest to report the impact of ASL interpretation on ischemic stroke assessment, particularly considering transit time and image quality effects that may mask true hypoperfusion.

## Materials and Methods:

This cross-sectional study was conducted in the Department of Radiodiagnosis, Santosh Medical College Hospital, Ghaziabad (NCR), India, over a period of 18 months. The study population included both male and female patients of all age groups and a total sample size of 60 patients was selected. Patients were enrolled if they presented within 24 hours of onset of neurological symptoms suggestive of acute ischemic stroke and if the patient or their relatives provided informed written consent for participation. Patients were excluded if they had contraindications to MRI such as ferromagnetic implants or severe claustrophobia, if they were uncooperative or extremely debilitated such that MRI acquisition was not feasible, or if they had a known idiosyncratic reaction/allergy to MR contrast agents. A total of 60 patients with clinical suspicion of acute ischemic stroke who were referred to the Department of Radiology and Imaging were evaluated irrespective of age or gender. MRI was performed on a 1.5 Tesla United Imaging MR system and image acquisition and reconstruction were carried out according to departmental standard operating procedures (SOPs) and reconstruction algorithms. Both non-contrast and contrast- enhanced MRI examinations were performed as per clinical requirements and lesion characteristics were assessed with emphasis on diffusion restriction and perfusion status to identify the infarct core and potentially salvageable tissue. The imaging protocol included routine structural sequences (T1-weighted and T2-weighted imaging), FLAIR, susceptibility-based imaging (SWI/T2* GRE) for hemorrhage detection, diffusion-weighted imaging with ADC maps (using multiple b-values) for early ischemia/core assessment and MR perfusion imaging (including arterial spin labeling where applicable) to quantify perfusion parameters such as cerebral blood flow and to evaluate diffusion-perfusion mismatch. MRI parameters were maintained as per standard sequence settings (including appropriate TR/TE and flip angles for T1, T2, FLAIR and DWI/ADC) to ensure uniformity across participants and MR findings were systematically analyzed and described for each case. Data were collected, entered into MS Excel 2010 and analyzed using Stata MP-17. The distribution of continuous variables was assessed using the one- sample Kolmogorov-Smirnov test to determine normality, after which parametric tests were applied for normally distributed data and non- parametric tests were applied for non-normally distributed data. Descriptive statistics were calculated for qualitative and categorical variables and graphical representation of variables was used to aid interpretation. Associations for categorical datasets were analyzed using the Chi-square test, while mean differences between two groups were assessed using the independent (Student) t-test as appropriate. Correlation analysis was performed to estimate the strength of relationships between two or more quantitative variables. A p-value <0.05 was considered statistically significant, whereas a p-value >0.05 was considered statistically insignificant.

## Results:

Table 1 (see PDF) showed that acute ischemic stroke was predominantly observed in older age groups. The highest proportion of patients belonged to the 61-70 years category, accounting for 16 patients (27%), followed by those older than 70 years with 13 patients (22%). The 51-60 years group also contributed substantially with 14 patients (23%). In contrast, younger age groups formed a smaller proportion of the study population, with 9 patients (15%) in the 41-50 years group, 5 patients (8%) in the 31-40 years group and only 3 patients (5%) below 30 years of age. Overall, the distribution indicated that 43 out of 60 patients (72%) were above 50 years, highlighting that the burden of acute ischemic stroke in this cohort increased markedly with advancing age. Table 2 (see PDF) demonstrated a male predominance among the study participants. Out of 60 patients, 36 were males (60%) and 24 were females (40%). This gender distribution suggested a higher representation of males presenting with acute ischemic stroke in the study setting. The male-to-female ratio was 1.5:1, indicating that for every 10 stroke patients, approximately 6 were male and 4 were female in the studied cohort. Table 3 (see PDF) described the arterial territory involvement and indicated that the middle cerebral artery territory was the most commonly affected. MCA involvement was noted in 39 patients (65%), reflecting the typical predominance of anterior circulation infarcts in acute ischemic stroke. These MCA strokes included cortical and subcortical infarcts, lacunar strokes, large territory infarcts and cases demonstrating penumbra. Posterior cerebral artery involvement was observed in 15 patients (25%), commonly corresponding to occipital infarcts, thalamic involvement and posterior circulation presentations such as visual symptoms. Anterior cerebral artery territory infarcts were the least frequent, seen in 6 patients (10%) and were mainly associated with medial frontal or paracentral infarcts as well as ACA-MCA watershed patterns. Table 4 (see PDF) evaluated DWI-PWI mismatch across stroke subtypes and territories, reflecting the presence of potentially salvageable penumbra. Overall, mismatch was present in 47 out of 60 patients (78.3%), indicating that a large majority of patients demonstrated diffusion-perfusion disparity suggestive of viable ischemic tissue beyond the established infarct core. In MCA territory strokes, mismatch was present in 32 of 38 cases (84.2%), with high sensitivity (92%) and specificity (85%), supporting the frequent presence of salvageable tissue in MCA infarcts and reinforcing the clinical utility of perfusion imaging for treatment planning in this group. In PCA territory strokes, mismatch was noted in 6 of 8 cases (75%), with sensitivity of 88% and specificity of 80%, indicating a moderate-to-high likelihood of penumbra, including extension to thalamic and midbrain regions. Similarly, ACA strokes showed mismatch in 3 of 4 cases (75%), with sensitivity of 85% and specificity of 90%, suggesting focal but clinically relevant mismatch in paramedian frontal and parietal regions. All lacunar or small vessel strokes (5 of 5 cases) showed mismatch (100%), with reported sensitivity of 100% and specificity of 75%, implying that perfusion deficits could extend beyond the small diffusion-restricted core even in small vessel disease. In contrast, venous infarcts (CVST) did not show mismatch in any of the 3 cases (0%), consistent with the observation that perfusion patterns in venous strokes may reflect congestion rather than a classical arterial penumbra. In hemorrhagic transformation cases, mismatch was present in 1 of 2 patients (50%), described as being limited to the peri-hematomal ischemic zone, suggesting that mismatch evaluation may still detect localized hypoperfusion adjacent to hemorrhagic components in selected cases. Table 5 (see PDF) summarized the diagnostic performance of MRI modalities and their statistical significance in acute ischemic stroke. DWI showed the highest diagnostic performance, with sensitivity of 100% and specificity of 90%, along with PPV of 96%, NPV of 100% and diagnostic accuracy of 98.3%. All 60 patients (100%) demonstrated restricted diffusion and the association was highly significant (χ^2^ = 60.00, p < 0.001), confirming DWI as the most reliable sequence for early infarct core detection in this study. ADC findings were positive in 59 patients (98.3%) and negative in 1 patient, with sensitivity of 98.3% and specificity of 92% and diagnostic accuracy of 96.7%. The results were also highly significant (χ^2^ = 56.06, p < 0.001), supporting ADC as a critical complementary tool to confirm true restricted diffusion and reduce the likelihood of misinterpretation due to T2 shine-through. MR perfusion (ASL/↓CBF) demonstrated sensitivity of 100% and specificity of 88%, with PPV of 94%, NPV of 100% and diagnostic accuracy of 95.0%. Perfusion abnormality was detected in all 60 cases (100%) and the statistical association was highly significant (χ^2^ = 60.00, p < 0.001), indicating that perfusion imaging consistently identified hemodynamic disturbances in this cohort, including subtle cases. DWI-perfusion mismatch showed sensitivity of 88.3% and specificity of 95%, with PPV of 97%, NPV of 89% and diagnostic accuracy of 91.7%. Mismatch was present in 48 patients (80%) and this relationship was highly significant (χ^2^ = 21.60, p < 0.001), emphasizing that mismatch assessment provided strong specificity for identifying salvageable tissue and therefore had important implications for therapeutic decision-making. (Table 6 - see PDF) presented the overall spectrum of MRI findings across sequences and reinforced the comprehensive contribution of DWI, ADC, MRA and ASL perfusion in acute ischemic stroke evaluation. T2/FLAIR hyperintensity within the involved vascular territory was identified in 57 patients (95%), often associated with mild mass effect in large infarcts, supporting its role in demonstrating established infarction or vasogenic edema and assisting with lesion age estimation when correlated with DWI. DWI detected restricted diffusion in all 60 patients (100%), reaffirming its role as the most sensitive method for early ischemia detection. ADC reduction corresponding to diffusion restriction was noted in 59 patients (98.3%), confirming cytotoxic edema and improving diagnostic confidence by distinguishing true infarct from T2 shine-through, while DWI-ADC mismatch was also observed in 59 patients (98.3%), consistent with early infarct evolution patterns. MRA demonstrated arterial narrowing or occlusion in 42 cases (70%), indicating that a substantial proportion had demonstrable large-vessel or clinically significant stenotic disease, which was relevant for identifying the vascular site of occlusion and guiding thrombolysis or thrombectomy planning. MRV detected venous sinus thrombosis in 3 cases (5%), reflecting a smaller subgroup with venous etiology and potential association with hemorrhagic conversion risk. MR perfusion-CBF abnormalities were present in all 60 patients (100%), indicating that perfusion imaging consistently demonstrated hypoperfusion and helped define infarct core and penumbra severity. The diffusion-perfusion mismatch (penumbra) based on CBF extent exceeding DWI lesion extent was noted in 31 patients (51.7%), indicating that about half the cohort demonstrated imaging evidence suggestive of potentially salvageable tissue by this specific measure. Chronic microangiopathy changes were observed in 35 patients (58%), suggesting a high background burden of small vessel ischemic disease, which could influence recovery potential and long-term prognosis. Hemorrhagic transformation was identified in 5 patients (8%) as blooming on GRE/SWI, indicating that a minority of cases showed hemorrhagic conversion, typically expected in larger infarcts or post-reperfusion contexts.

Figure 1 (see PDF) displays MRI brain images showing multiple acute non-haemorrhagic infarcts involving the right fronto-parietal, left parietal, bilateral occipital, left frontal and bilateral cerebellar regions. MRI brain shows multiple acute non-haemorrhagic infarctsinvolving the right fronto-parietal, left parietal, bilateral occipital, left frontal and bilateral cerebellar regions, with corresponding DWI restriction and low ADC, consistent with acute ischemia. Background findings include chronic small vessel ischemic changes (Fazekas III) and age-related atrophy. ASL perfusion demonstrates multifocal marked CBF reduction with limited perifocal preservation, indicating predominantly completed infarcts with minimal salvageable penumbra. Findings are consistent with multi- territory acute ischemic stroke on a background of chronic microangiopathy. MR angiography is advised and contrast avoidance is recommended due to AKI, with management focused on antiplatelet therapy after renal stabilization, statin therapy and hydration. Figure 2 (see PDF) presents GRE and ASL CBF images, complementing the findings in Figure 1 (see PDF) by showing perfusion abnormalities in the affected regions. In Figure 3 (see PDF), the MRI reveals an acute hemorrhagic infarct in the right PICA territory, which causes mass effect on the fourth ventricle, resulting in mild bilateral lateral ventricular dilatation. MRI brain reveals an acute hemorrhagic infarct in the right PICA territory, causing mass effect on the fourth ventricle with mild bilateral lateral ventricular dilatation. DWI shows diffusion restriction and ASL perfusion demonstrates reduced CBF in the same region, with no significant DWI-ASL mismatch, indicating predominantly completed infarct with minimal salvageable penumbra. MRA/MRV is unremarkable and chronic small vessel ischemic changes (Fazekas grade I) are noted in bilateral frontal and left parieto-temporal white matter. Management includes neuroprotective measures, careful blood pressure control, supportive hydration and antiplatelet therapy once hematoma stability is confirmed, with surgical intervention reserved for worsening mass effect or hydrocephalus. Figure 4 (see PDF) shows the acute haemorrhagic infarct in the right PICA territory, with associated mass effect on the fourth ventricle and mild ventricular dilatation.

## Discussion:

In the present study, acute ischemic stroke clustered in the older decades, with 43/60 patients (72%) aged >50 years and nearly half (29/60; 49%) aged >60 years; the largest single group was 61-70 years (16/60; 27%), followed by >70 years (13/60; 22%). This age pattern is consistent with population-level observations that stroke occurs predominantly in later life; for example, Appelros *et al.* (2009) [13] reported a higher average age at stroke onset in women than men (mean 72.9 vs 68.6 years), reinforcing that increasing age strongly shifts the clinical burden toward older groups, similar to the skew seen in our hospital cohort. A male predominance was observed in our cohort (36/60; 60%), yielding a male-to-female ratio of 1.5:1. Comparable male excess has been documented in Indian multicenter data Sylaja *et al.* (2017) [14] reported 67.2% men among ischemic stroke patients (mean age 58.3±14.7 years), which is directionally similar to our 60% male distribution, although our proportion was modestly lower, plausibly reflecting local referral patterns and inclusion of both arterial and venous etiologies in a single imaging-based cohort. Territorial analysis in our study showed MCA dominance (39/60; 65%), with higher posterior circulation representation (PCA 15/60; 25%) and smaller ACA contribution (6/60; 10%). When compared with vascular-territory datasets derived from imaging-defined large vessel patterns, Nichols *et al.* (2021) [15] reported MCA territory as the most frequent distribution (62.3%), while PCA (12.1%) and ACA (6.6%) were lower than in our cohort; the relatively higher PCA and ACA proportions in our study may reflect case-mix differences (including posterior circulation symptom-driven referrals and smaller territorial infarcts captured early on MRI) and the inclusion of varied stroke mechanisms within a fixed sample size. Diffusion-perfusion mismatch, used as a surrogate for potentially salvageable penumbra, was common in our cohort (47/60; 78.3%), particularly in MCA territory strokes (32/38; 84.2%), supporting the added value of perfusion imaging for treatment-relevant tissue profiling. In contrast, Lansberg *et al.* (2007) [16] reported perfusion-diffusion mismatch in 54% of the DEFUSE population using predefined mismatch criteria; the higher mismatch proportion in our study could relate to differences in enrollment window (within 24 hours in our study vs narrower hyperacute trial windows), patient selection (routine clinical referrals rather than trial-eligible candidates) and heterogeneity of stroke subtypes where perfusion deficits may extend beyond small diffusion cores. Our diagnostic performance results emphasized DWI as the most sensitive sequence: restricted diffusion was present in 60/60 patients (100%), while ADC reduction confirming true restriction was present in 59/60 (98.3%), producing very high diagnostic accuracy (DWI 98.3%; ADC 96.7%). These findings closely align with foundational comparative work by van Everdingen *et al.* (1998) [17], who demonstrated that DWI detected 98% of ischemic lesions (91% on FLAIR and 71% on early T2-weighted imaging), supporting the concept that DWI is the most robust early marker of infarct core, with ADC providing confirmatory value against T2 shine-through-mirroring the complementary DWI-ADC behavior in our dataset. Perfusion imaging in our study detected hemodynamic abnormality in all cases (60/60; 100%) with high diagnostic utility (sensitivity 100%, specificity 88%, accuracy 95%) and mismatch-based assessment showed strong specificity for salvageable tissue (specificity 95%). In contrast, Nael *et al.* (2013) [18] observed that ASL did not demonstrate any perfusion abnormality in 11% of patients and reported agreement between ASL and DSC on type/location of perfusion abnormality in 71% and 80% of cases, respectively; our higher abnormality detection rate likely reflects that our cohort comprised clinically suspected acute stroke cases with MRI-confirmed infarction (DWI positive in 100%), whereas ASL performance can be limited by transit-delay effects, image quality variability and the challenge of distinguishing hypoperfusion from delayed arrival without complementary maps. On vascular imaging, MRA demonstrated arterial narrowing/ occlusion in 42/60 patients (70%), indicating a substantial burden of demonstrable steno-occlusive disease in clinically suspected acute ischemic stroke referrals, which is clinically relevant for thrombolysis/thrombectomy triage and explaining the high mismatch rates in major-territory infarcts. Methodologically, the reliability and limitations of TOF-MRA for intracranial steno-occlusive disease have been evaluated against reference standards; Choi *et al.* (2007) [19] assessed high- resolution 3D TOF-MRA for intracranial atherosclerotic steno-occlusive disease using DSA as reference, supporting its diagnostic role while acknowledging technical factors that can influence grading contextualizing why our MRA "positivity" rate should be interpreted as clinically significant detection rather than an exact stenosis quantification substitute for catheter angiography. Finally, background small-vessel disease and hemorrhagic evolution were notable in our MRI spectrum chronic microangiopathy was present in 35/60 patients (58%), while hemorrhagic transformation was seen in 5/60 (8%) on GRE/SWI, suggesting that a sizable proportion carried pre-existing ischemic brain vulnerability but only a minority demonstrated early hemorrhagic conversion. In an elderly thrombolysis-focused cohort, Liu *et al.* (2020) [20] reported severe leukoaraiosis in 26.4% and hemorrhagic transformation in 12.0%; compared with that, our hemorrhagic transformation rate was lower (8%), plausibly because our cohort was not restricted to thrombolysed patients (where bleeding risk is higher) and because hemorrhagic conversion may evolve over time, while our microangiopathy prevalence (58%) was higher partly because MRI (including FLAIR) is more sensitive to mild-to-moderate white matter disease than CT-based grading and our definition included Fazekas I-III rather than "severe" only.

## Conclusion:

We found that diffusion-weighted imaging (DWI) and ADC mapping effectively identify infarct cores in acute ischemic stroke, boosting diagnostic confidence. MR perfusion added valuable hemodynamic insights to highlight hypoperfused tissue and diffusion-perfusion mismatch, indicating salvageable areas. The integration of DWI, ADC and perfusion data improved lesion characterization, supporting more informed clinical decisions.
